# Structural dynamics of human deoxyuridine 5’-triphosphate nucleotidohydrolase (dUTPase)

**DOI:** 10.1038/s41598-024-76548-x

**Published:** 2024-10-30

**Authors:** Ravdna Sarre, Olena Dobrovolska, Patrik Lundström, Diana Turcu, Tatiana Agback, Øyvind Halskau, Johan Isaksson

**Affiliations:** 1 Department of Chemistry, UiT the Arctic University of Norway, PO Box 6050, Stakkevollan, 9037 Langnes, Tromsø, Norway; 2 Department of Biological Sciences, University of Bergen, Box 7803, 5020 Bergen, Norway; 3 Department of Physics, Chemistry and Biology, Linköping University, 581 83 Linköping, Sweden; 4 Department of Molecular Sciences, Swedish University of Agricultural Sciences, P.O Box 7070, 750 07 Uppsala, Sweden; 5 Department of Pharmacy, UiT the Arctic University of Norway, PO Box 6050, Stakkevollan, 9037 Langnes, Tromsø, Norway

**Keywords:** Human dUTPase, Protein dynamics, Multidimensional NMR, Solution-state NMR, Enzyme mechanisms

## Abstract

Structural- and functional heterogeneity, as well as allosteric regulation, in homo-monomeric enzymes is a highly active area of research. One such enzyme is human nuclear-associated deoxyuridine 5’-triphosphate nucleotidohydrolase (dUTPase), which has emerged as an interesting drug target in combination therapy with traditional nucleotide analogue treatment of cancer. We report, for the first time, a full structural dynamics study of human dUTPase by NMR. dUTPase has been investigated in terms of structural dynamics in its *apo* form, in complex with the modified substrate resistant to hydrolysis, 2’-deoxyuridine 5’-α,β-imido-triphosphate (dUpNHpp), as well as the product, 2’-deoxy-uridine-monophosphate (dUMP). The *apo* form of the enzyme displayed slow dynamics in the milli- to microsecond regime in relaxation dispersion experiments, which was further slowed down to observable heterogeneity upon substrate-analogue binding. The results suggest that the non-hydrolysable substrate-analogue traps the enzyme in the conformational isomerization step that has been previously suggested to be part of the enzyme catalysis kinetics cycle. The observed heterogeneity fits well with the pattern expected to emerge from the suggested kinetic model, and no evidence for homotropic allosterism was found. The heatmaps of the slow dynamics, chemical shift perturbation upon substrate binding and conserved regions of the enzyme sequence all displayed a similar pattern, which suggests that the structural dynamics is finely tuned and important for the biological function of the enzyme for binding, conformational shift, catalysis and substrate release.

**Supplementary Information:**

The online version contains supplementary material available at 10.1038/s41598-024-76548-x.

## Introduction

Deoxyuridine 5’-triphosphate nucleotidohydrolase (dUTPase; EC 3.6.1.23) catalyzes the hydrolysis of deoxyuridine 5’-triphosphate (dUTP) into deoxyuridine 5’-monophosphate (dUMP) and pyrophosphate (PPi) in all free-living organisms, including viruses^1^. It is essential for life to maintain a low dUTP:dTTP (deoxythymidine 5’-triphosphate) ratio^2^ and failure to do so results in chromosome fragmentation due to Uracil misincorporation and thymineless cell death. This makes species-specific dUTPase a potentially attractive pharmaceutical target against a number of pathogens, including malaria^3^ and tuberculosis^4^, where the biosynthesis of dTMP relies exclusively on the dUTPase activity.

dUTPase is also a potential drug target for many cancers. Studies on a drug resistant human bone-marrow-derived cell line (U-2 OS) suggest that dUTPase levels may influence the aggressiveness of osteosarcomas^5^. In patients, elevated expression of dUTPase is negatively correlated with clinical response to 5-FU therapy^6^. Thus, elevated dUTPase activity limits the therapeutic efficacy of the chemotherapy both by decreasing the intracellular dUTP pool and by increasing the levels of dUMP. Increased dUMP restores the dTTP:dUTP balance needed for cell proliferation through the action of Thymidylate synthase (TS). Suppression of human dUTPase is therefore highly desirable in combination with conventional cancer treatment. A report of novel nanomolar dUTPase inhibitors has presented the first in vivo (MX-1 breast cancer xenograft model in mouse) evidence that human dUTPase inhibition enhances the effect of TS inhibitors^7,8^.

**Fig. 1:**
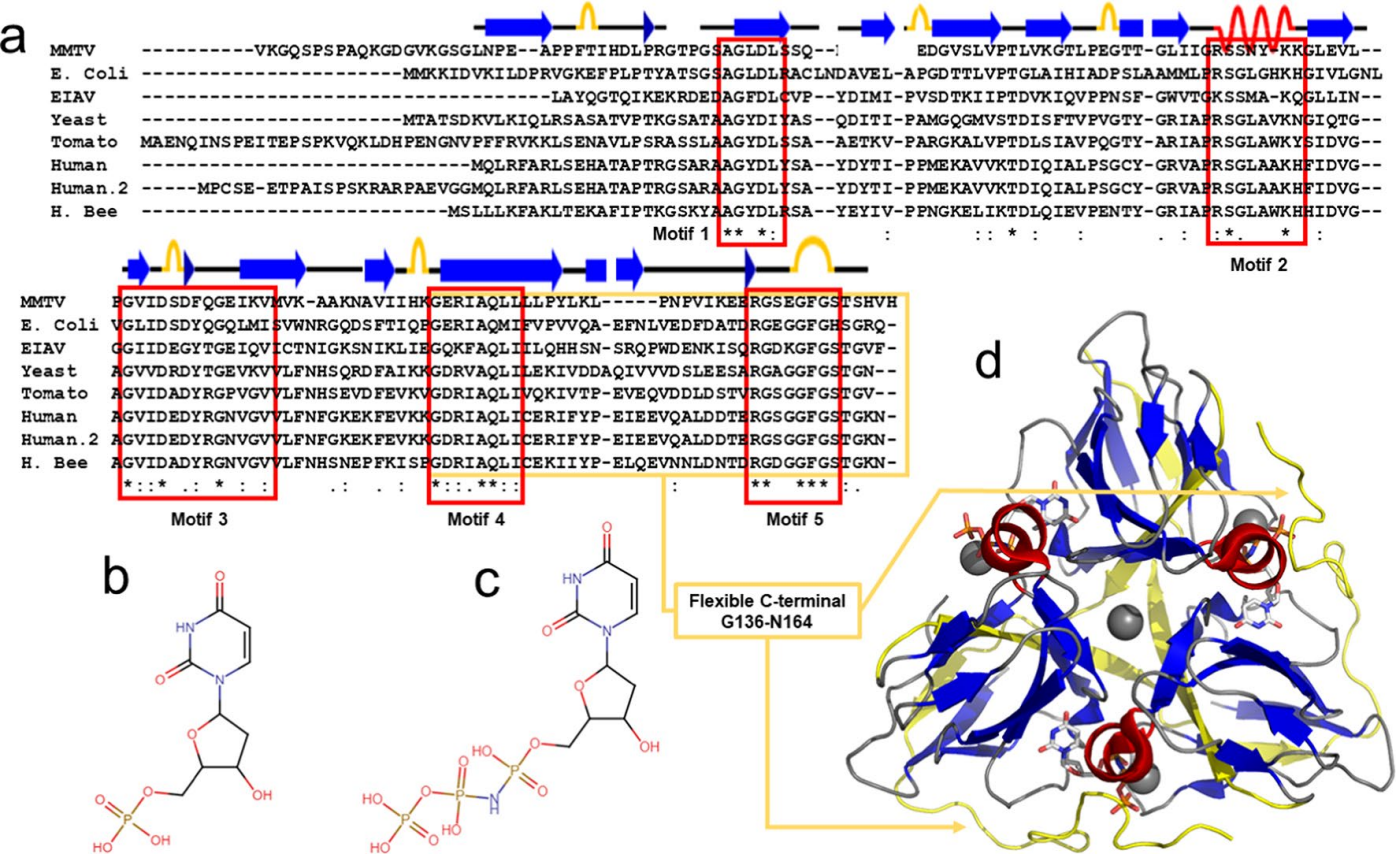
(**a**) Multiple sequence alignment of dUTPase using Clustal D with default parameters. Conserved amino acids are denoted by ‘*’, conserved motifs are indicated by red boxes, as are approximate positions of β-strands (blue arrows), α-helices (pink coils), and turns (yellow arcs). The flexible C-terminal is denoted by the yellow box. Chemical structures of (**b**) 2’-deoxyuridine-5’-monophosphate (dUMP), (**c**) 2’-deoxyuridine-5’-α,β-imido-triphosphate (dUpNHpp). (**d**) The structure of trimeric dUTPase (cartoon representation), its substrate analogue dUDP (stick representation), and coordinated Mg^2+^ (dark gray sphere representation). Chemical structures of dUMP and dUpNHpp were drawn using Chemical Sketch Tool (rcsb.org/chemical-sketch), and panel c) is rendered in PyMOL using pdb: 2HQU, Human dUTPase in complex with dUDP. The last residue in two of the monomers is N164, and the entire flexible C-terminal arm (G136-N164 is not resolved in this crystal structure.

Human dUTPase is a homotrimer forming three identical active sites in the interface between them, and it contains five highly conserved regions (Fig. 1)^9^. The dUTPase binding pocket is highly specific for uracil and the phosphate chain coordination utilizes Mg^2+^, analogous to that of DNA polymerases. Both *Drosophila-*^10,11^ and human^12,13^ dUTPase have been shown to undergo a conformational rearrangement of the C-terminus upon substrate binding. Ordering of the otherwise flexible C-terminal arm of the protein forms a cap over the active site and is essential for its function.

Early reports suggested that the three active sites per trimer to be independent in *Escherichia coli*^14,15^. In contrast, NMR titrations and kinetic data for *Drosophila*proposed a significant deviation from the model of independent active sites within the homotrimer, leading them to suggest allosterism in eukaryotic dUTPase^10^. A more recent paper^16^, however, elegantly used hybrid mutated hetero-trimer dUTPase assemblies to demonstrate that the individual active sites remained functional when one or two other active sites were rendered inactive by point- or deletion mutations. This finding suggests that strong allosteric regulation between the active sites of the homotrimer is unlikely. Hence, there has been some controversy regarding allosterism in eukaryotic dUTPases, motivating further studies of the enzyme dynamics at a residue-specific resolution.

To date, most crystal structures of dUTPase in complex with different ligands do not resolve the flexible residues in the C-terminus, and the ability to grow high quality crystals in general appears to be ligand dependent^7,8,13,17,18^. A general observation is that only ligands highly similar to the native substrate are able to order the C-terminal arm in crystal structures, possibly because many stabilizing interactions involve the phosphate(s) of the substrate/product and structural metal ions.

The size of the dUTPase complex of ~ 50 kDa, together with the internal dynamics in the milli- to microsecond regime, limits the spectral quality if deuterated samples and TROSY type experiments are not utilized. For this reason, the only previously published NMR study has been limited to a few resonances belonging to the flexible C-terminal tail residues of *Drosophila*dUTPase that are readily identifiable^10^. The successful assignment of the backbone of the enzyme, previously published by us^19^, enabled the full residue-specific dynamics study of human dUTPase. In this study we present the structural dynamics of each residue on the dUTPase backbone, on the slow (ms-µs) and fast (ns-ps) NMR time scales, and relate it to the biological function of the enzyme as well as previously published studies using other methods. To achieve this, we employ several experiments to characterize structural dynamics by NMR spectroscopy. The general order parameters sensitive to the nanosecond regime are fit to a series of experiments measuring R_1_ relaxation, R_2_ relaxation and the steady-state NOE^20^. To assess the millisecond to microsecond regime of dynamics, relaxation dispersion experiments are utilized^21–23^. This class of experiments has since its introduction revolutionized the kind of information that can be obtained related to the biological function of biomolecules, which often takes place on the millisecond to microsecond time scale. Furthermore the hydration of the enzyme is characterized, both in terms of water exchange with the amide hydrogens through CLEANEX-PM^24^ and water molecules with long residence times on the enzyme surface through ^15^N edited,^14^N/^12^C-filtered ROESY^25^.

## Experimental

### Expression

Residues 24–164 of human dUTPase (UniProt identifier P33316-2) with an N-terminal His_6_ tag were cloned into expression vector pET11a using BamHI and NdeI as restriction enzymes. Protein was expressed in *Escherichia coli* expression strain BL21(DE3)Star (Life Technologies). For expression of ^15^N/^13^C/^2^H-labeled dUTPase, a minimal medium based on M9 medium was prepared in D_2_O and ampicillin was used as the antibiotic. Expression of dUTPase was done at 37 °C; induction of expression using IPTG was done at OD_600_ = 1.3 and cells were harvested 12 h after induction. Protein was purified by IMAC followed by gel filtration at pH 7.5 and using 2 mM DTT in the buffers as a reducing agent. The NMR samples contained approximately 1 mM protein in 10 mM NaPi (pH 7.0), 50 mM NaCl, 5 mM MgCl_2_, 0.5 mM tris(2-carboxyethyl)phosphine (TCEP), 0.01% NaN_3_ in 10%:90% D_2_O:H_2_O. Ligand was added in a 1.2-2.0:1 ratio.

### NMR spectroscopy

NMR experiments used for assignment of the dUTPase and its relaxation were acquired on Bruker Avance III HD spectrometers equipped with inverse triple resonance TCI probes with cryogenic enhancement for ^1^H, ^13^C and ^2^H, operating at 600 and 850 MHz for ^1^H respectively. Complementary data was also acquired on a 600 MHz Varian Inova spectrometer equipped with an inverse triple resonance HCN probe with cryogenic enhancement for ^1^H.

Spectra were collected at 286, 298 and 310 K. The gradient sensitivity enhanced 3D triple-resonance TROSY versions of the pulse sequences HNCA, HN(CO)CA, HNCO, HN(CA)CO, HNCACB were acquired and processed using Topspin 3.5pl7 and/or NMRPipe. All assignment and spectrum analysis was subsequently done in CARA, version 1.8.4.2^26^. Relaxation experiments of T1-, T1tho, T2-, NOE- and relaxation dispersion ^15^N-TROSY-HSQC were acquired using VnmrJ and Topspin 3.5pl7 for the respective systems. The relaxation dispersion pulse sequence on the Varian system as acquired using a pulse sequence provided by Prof. Lewis Kay, and on the Bruker instruments a version adopted from the Kay sequence by Maksim Maysel at Bruker BioSpin (hsqcrexetf3gpsitc3d.3). For the hydration study, the 2D CLEANEX-PM^24^ (30, 60 and 100 ms mixing) and 3D X-filtered/edited ROESY^25^ (60 ms spinlock) were acquired.

All data sets were processed using the NMRPipe system^27^ and Topspin 3.5pl7. Integration and curve fitting was performed in PINT v2.1.0^28^, Nessy 12.3.1^29^, Modelfree 4.0^30^ and Relax 5.0.0^31,32^, the latter three using the NMRBox resource^33^.

### Multiple sequence alignments

Multiple sequence alignments of dUTPase protein sequences from a selection of organisms were performed using Clustal Omega and default settings^34,35^. The origin organisms were picked to correspond to the species used for a similar alignment in^36^, with the addition of *Homo sapiens*, isoform 2, the subject of this work, and *Apis mellifera* to introduce a representative from the arthropod phylum. The final list of sequences was *Escherichia coli*, strain K12 (E. coli, P06968), *Mouse mammary tumor virus*, strain BR6 (MMTV, P10271), *Homo sapiens* isoform 2 (Human.2, P33316-2), *Saccharomyces cerevisiae*, strain ATCC 204,508/S288c (Yeast, P33317), *Solanum lycopersicum* (Tomato, P32518), *Apis mellifera* (Honeybee, A0A7M7SRM3), and Equine infectious anaemia virus, isolate 1369 (EIAV, P11204).

## Results and discussion

Previously published results suggested that the structural dynamics of human dUTPase is intimately linked to its biological function: The flexible C-terminal arm is necessary for catalytic activity, as the deletion mutant is inactive, see Fig. 1 and Ref^16^. The closed form has been shown to close the active site, restrict water access by stacking F158 over the substrate and participate in the coordination of the tri-phosphate^13^. Detailed x-ray analysis using the modified substrate, dUpNHpp, capturing the slowed down reaction in multiple conformations^17^, together with enzyme kinetic studies, has further suggested a slow but not rate limiting structural isomerization step in the 20 Hz time scale prior to catalysis^12^.

### *Apo* form enzyme dynamics

**Fig. 2:**
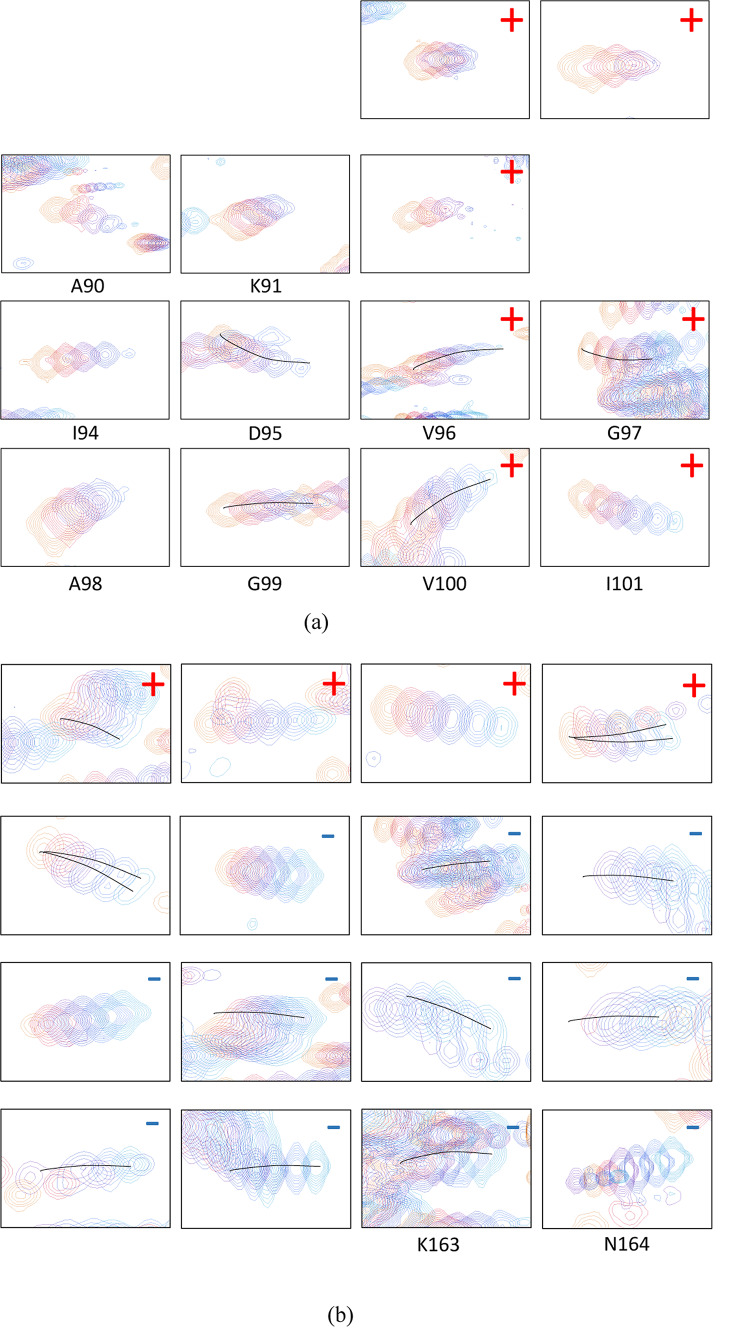
(**a**) Temperature dependence of residues L88-I101 of the *apo* protein, in the region of the conserved motifs 2–3. (**b**) Temperature dependence of residues 149–164, in the C-terminal arm. Residues that display increased intensities upon increasing the temperature have been labelled with **+**, and correspondingly, residues displaying decreased intensities upon increasing the temperature have been labelled with **−**.

The structural dynamics was first assessed by recording the temperature dependence of the chemical shifts of the *apo* form of the enzyme. ^15^N-HSQC spectra were recorded at 3 K intervals between 277 and 310 K and superimposed. Examples of the temperature induced chemical shift drifts of the residues of the conserved motifs 2–3 and for the C-terminal arm are shown in Fig. 2.

**Fig. 3:**
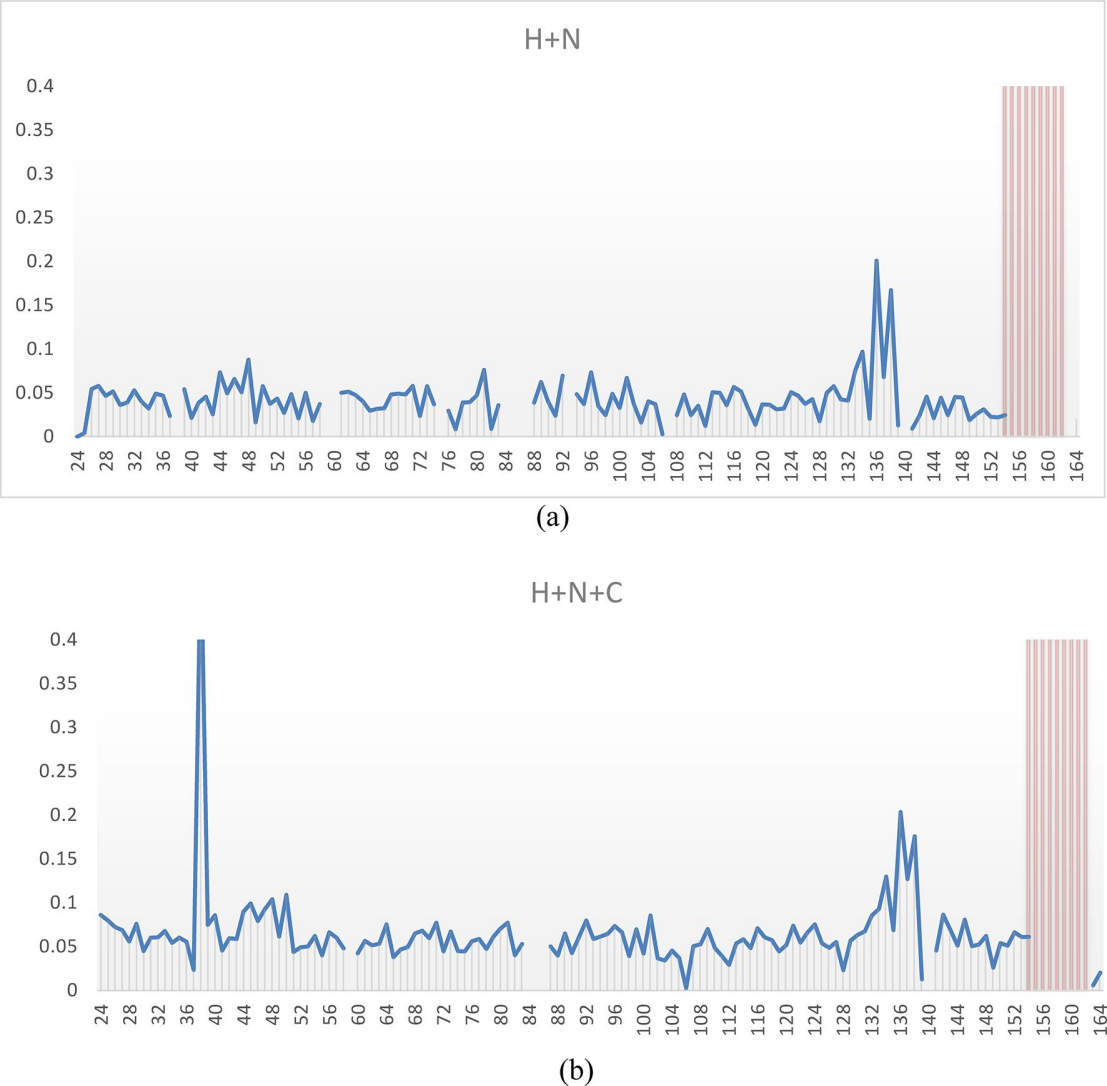
Chemical shift perturbation of the (**a**) H and N, or (**b**) the H, N and C when increasing temperature from 25 to 37 °C. Red bars indicate that the resonances were undetectable at the lower temperature.

The temperature dependent behavior of the NH resonances highlights that we have indeed structural dynamics in several time scales for the *apo* form. For residues E152-R153 the resonances appear to split into two individually detectable populations at lower temperatures, that coalesce close to 300 K. For many residues in the C-terminal arm, for example G154, G156, G157, F158, G159, T160 and T161, the intensity of the resonances decrease as the temperature is *increased*. In contrast, for residues near motives 2–3, for example L88, A89, A90, K91, H92, I94 and D95, the signal intensities instead decrease as the temperature is *decreased*. Other residues are unassignable close to these regions, most notably residues 84–87 are not possible to detect or assign in any studied preparation of the enzyme, including different ligands and different temperatures. These observations confirm the presence of substantial slow motions, above-, at-, below- and across the NMR time scale for the *apo* form of human dUTPase. The weighted cumulative effect of the chemical shift perturbation as a function of temperature is reported in Fig. 3, using the square root of the sum of the chemical shift perturbation square averages, using a scaling factor, α, of 0.14 for the amide nitrogen atoms and 0.35 for the carbonyl carbons on the backbone as estimated from their average standard deviations in the BMRB chemical shift database.

**Fig. 4:**
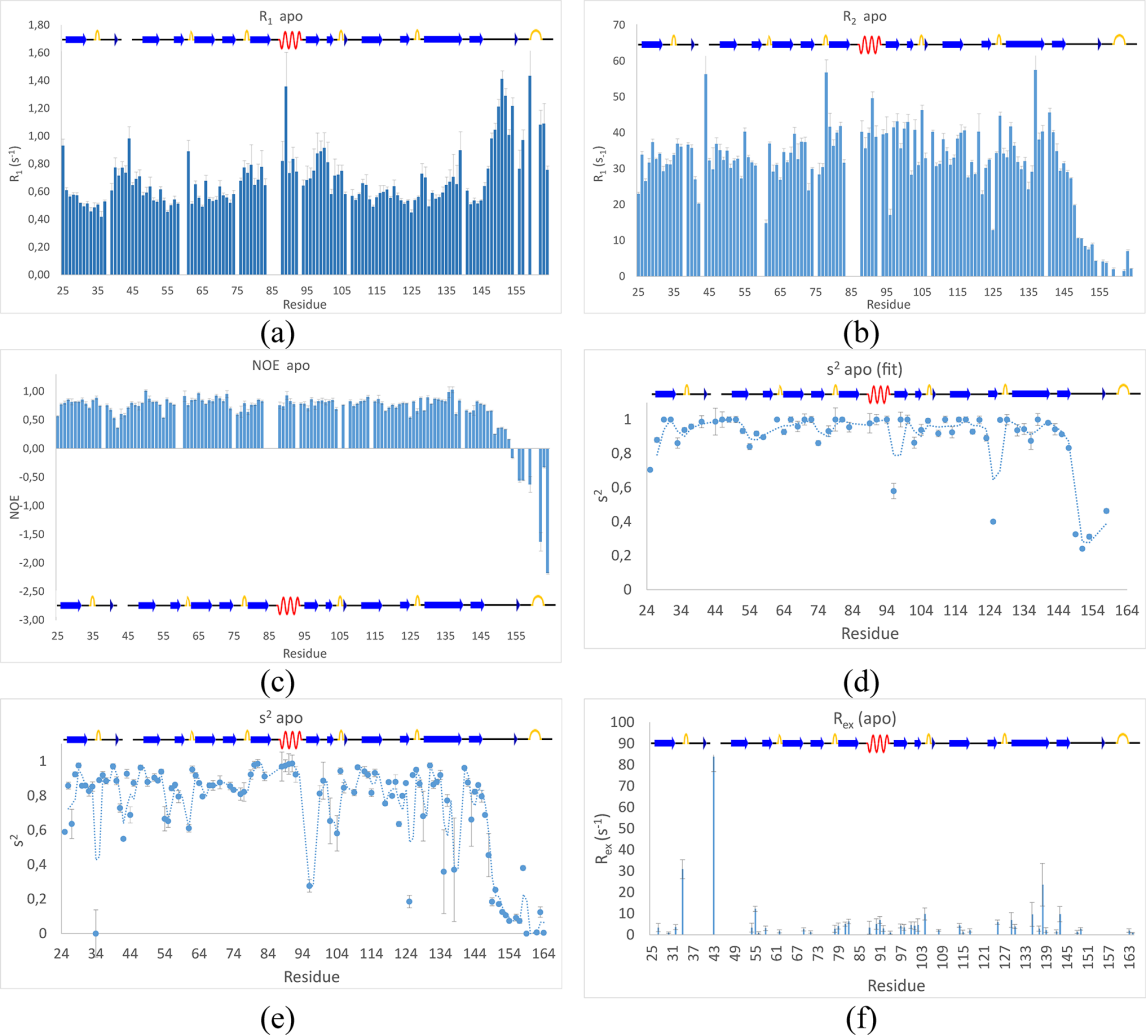
Relaxation parameters, (**a**) R_1_, (**b**) R_2_, (**c**) ^15^N-NOE, (**d**) S^2^ calculated with Model free at 600 MHz fitting S^2^ and t_e_, (model 2 in the Model free documentation) (**e**) S^2^ calculated with Relax at 600 + 850 MHz fitting S^2^, t_e_ and R_ex_ (model 4 in the Modelfree documentation) and (**f**) R_ex_ calculated from relaxation dispersion at 600 MHz. All experiments were acquired at 37 °C.

To get more insight into the structural stability of the *apo* form enzyme and evaluate the ^1^H-^15^N amide bond order parameters (S^2^), the R_1_, R_1ρ_ and R_2_ relaxation rates, together with the steady-state ^15^N-NOE were determined and data presented on Fig. 4. The C-terminal arm of the *apo* form, housing the strictly conserved Motif 5 (Fig. 1, and Ref^37^), displayed complex dynamics, involving several different time scales.

Most notably, there is a pronounced increase in the R_1_ and a corresponding decrease in R_1ρ_/R_2_ for residues 148–164 compared to the well-folded parts of the protein complex, which reflects an increased flexibility and fast internal motion in the nano- to picosecond scale of the C-terminal arm (Fig. 4a,b). The flexibility can also be observed by the steady-state NOE parameters, that drop from close to 1 for the well folded regions to negative values for the fully flexible residues of the C-terminal arm. The disordered nature of the C-terminal has earlier been qualitatively observed with both NMR^10^ and crystallography^38^, but herein its disorder is for the first time quantitatively described in solution. The increased flexibility of the C-terminus is reflected as a sharp drop in the fitted generalized order parameters, s^2^ (Fig. 4d,e).

In the *apo* form, negative ^15^N-NOE were confirmed for residues G154, G156, G157, G159, G162, K163 and N164, meaning that these residues are highly flexible, having effective correlation times reminiscent of a small molecule in solution rather than inheriting the slow tumbling of the protein complex with a rotational correlation time of approximately 28 ns (Fig. 4c). It should be noted that the interspersed residues in the C-terminal stretch from L148 to N164 produced weak resonances that were difficult to integrate accurately. Together with the temperature dependent observations above, we conclude that the C-terminal arm is highly flexible in terms of internal rotations. Importantly, the spectral properties of the C-terminal are also greatly affected by the arms participation in a slow, more concerted motion in the millisecond regime, plausibly an opening and closing of the C-terminal arm relative to the core of the enzyme.

**Fig. 5:**
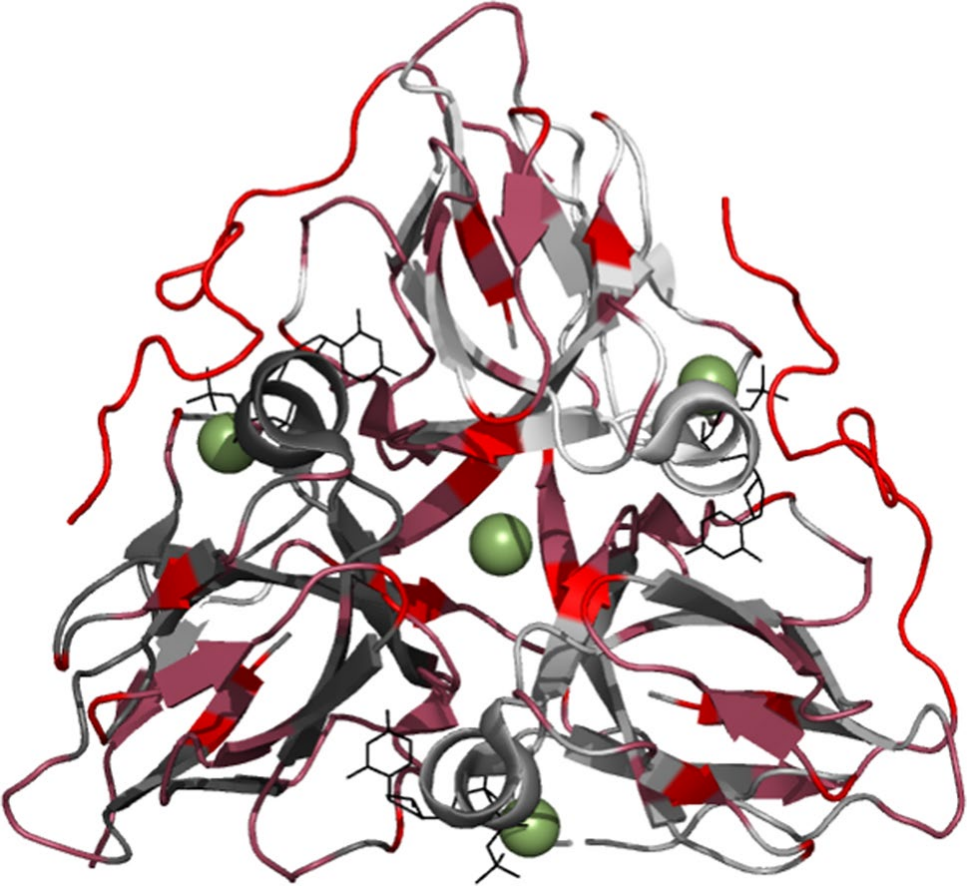
A heatmap of the dUTPase residues displaying the lowest order parameters. Bright red: S^2^ < 0.5, dark red: 0.5 < S^2^ < 0.8. (Pdb: 2HQU).

There is a more subtle but noteworthy increase in both R_1_ and R_1ρ_/R_2_ for the stretch of residues between 78 and 104, who are involved in substrate recognition (including the strictly conserved Motif 2 and half Motif 3). There is also a moderate increase in R_1_ for residues 40–46 (including the strictly conserved Motif 1). The increase in R_1_ generally indicates increased motions in the nanosecond regime affecting the order parameters while there is a simultaneous increase in R_1ρ_/R_2_ (instead of a decrease which would have been expected for a highly flexible region). This leads to the conclusion that there are additional motions that contribute to the spectral density functions in the nanosecond regime with additional slow motions closer to the NMR time scale (in the millisecond regime), that contribute to line broadening (and R_2_). This is supported by the absence of response in the ^15^N-NOE parameter for these regions^39^. A heatmap highlighting the most disordered parts of the enzyme is presented in Fig. 5. Interestingly, this disorder is not restricted to motifs directly involved in substrate binding.

The complex dynamics of the enzyme was expected to manifest in relaxation dispersions, an experiment that is sensitive to exchange processes in the millisecond regime. Relaxation dispersion data acquired at 600 and 850 MHz did indeed display slow dynamics for the backbone of a large number of residues. Analysis in Nessy, using both datasets simultaneously, achieved the best fit to a slow dynamics model (model 7 in the Nessy documentation) over the model devoid of slow dynamics (model 1 in the Nessy documentation) for 46 residues evenly distributed over the sequence, out of the 122 residues that could be reliably integrated. A slightly more conservative analysis of only the 600 MHz dataset, which was the highest quality dataset, is reported in Fig. 4f, and 34 relaxation dispersion profiles are displayed in Figure S3 in the Supporting Information with a P-value cut-off of 0.1. The relaxation dispersion data was acquired at two field strengths and one temperature, and should be interpreted conservatively to avoid overfitting. Hence we focus on the qualitative interpretation of which residues display the strongest relaxation dispersion effects. The highest quality dispersion profiles are obtained for residues E141 and I142, which is in the beta sheet where the flexible C-terminal arm connects the core structure. The full heatmap for the relaxation dispersions are presented in Fig. 8D, and it highlights that large parts of the enzyme is affected by dynamics in the milliseconds to microseconds regime, with a qualitative over-representation near the active site and on beta sheet regions.

An attempt was made to correlate the chemical shift difference between the resting state and the “excited minor state” acquired from fitting the dispersion profiles, with the chemical shift difference between the apo form and the holo form. If the minor state of the apo form had indeed been owed to a conformation that is pre-organized for substrate binding, one could expect some correlation between the Dd from the dispersion profile fit and that from the chemical shift perturbation of binding. These correlations were weak with R^2^ = 0.47 for dUMP and R^2^ = 0.39 for dUpNHpp (Figure S4 in the Supporting Information). There is however also a substantial direct contribution to the chemical shift of the holo-form from the ligand-enzyme interactions, and therefore a good correlation may not be expected even if the minor state is indeed originating from conformational selection upon binding. We therefore would like to refrain from making any conclusions regarding the nature of the minor conformational state(s).

In conclusion, the dynamic picture of the *apo* form enzyme reveals an enzyme that is relatively active in pulsating and “breathing” motions, adopting the conformation of the open active site for substrate binding.

### Substrate binding

**Fig. 6:**
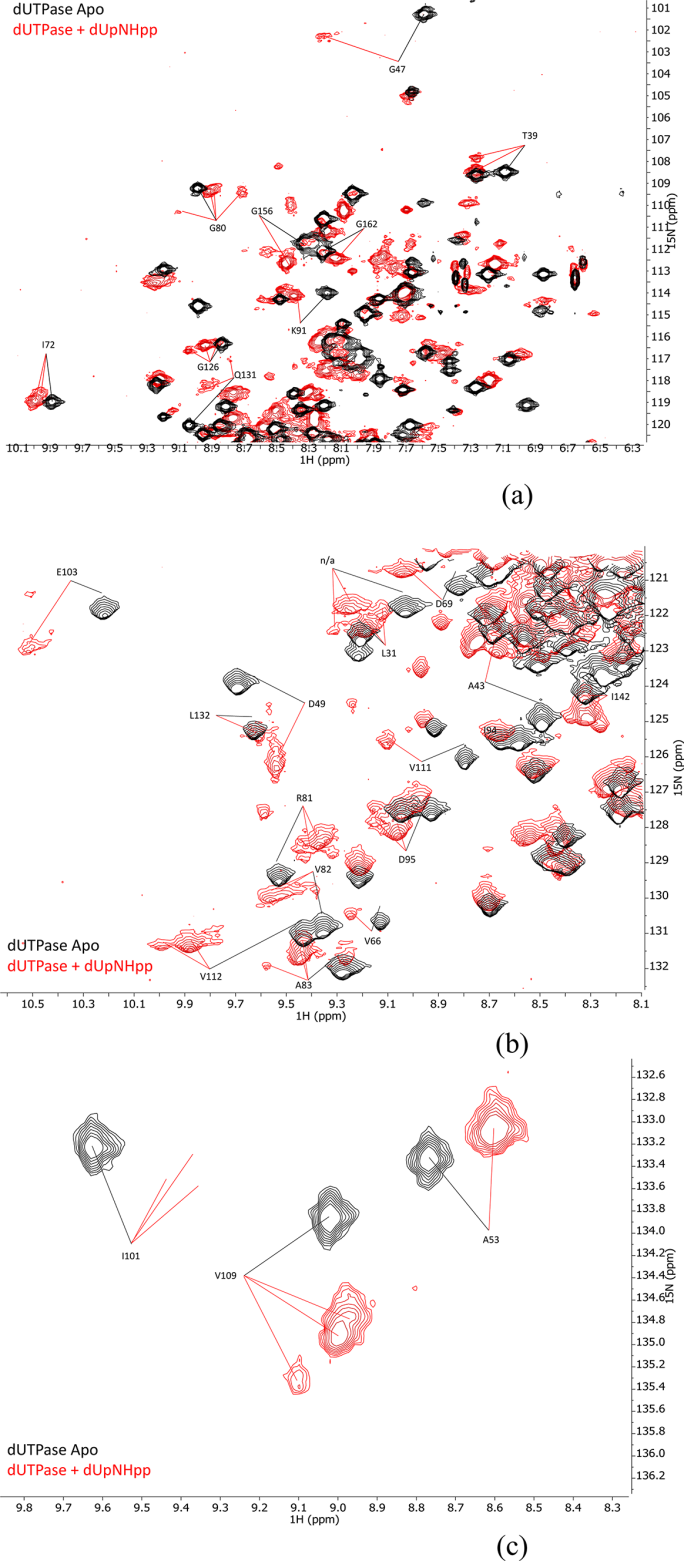

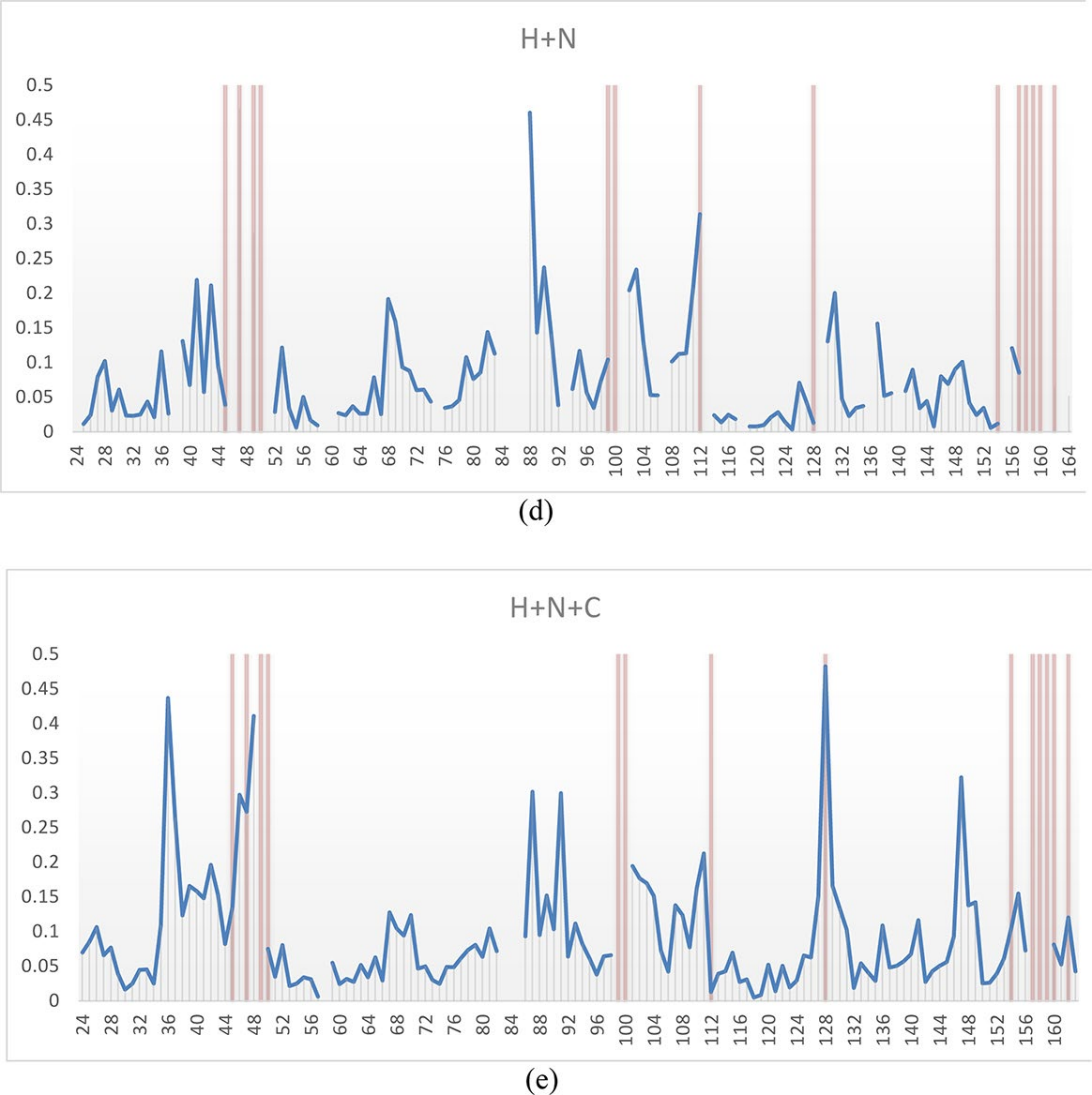
(**a**-**c**) Superimposed ^15^N-HSQC spectra of dUTPase (black) and dUTPase + dUpNHpp (red) showing both the induced chemical shift differences caused by the binding event, as well as the change in lineshape and heterogeneity. (**d**) and (**e**) show the cumulative chemical shift perturbation upon substrate (dUpNHpp) binding where the red bars indicate that the chemical shift of the *holo* form was not producing a detectable resonance.

The addition of 1.2 molar equivalents of the modified “uncleavable” substrate, dUpNHpp, results in dramatic changes to the spectral quality as well as the chemical shifts of the protein resonances (Fig. 6a-d). The heterogenic change is not concentration dependent, and addition of up to 4 molar equivalents does not further affect the spectrum (Figure S1 in the SI). This observation indicates that the heterogeneity does not arise from binding events, as any state with unsaturated binding sites would be expected to be affected by the ligand concentration even in the case of strong negative cooperativity between the active sites. Such strong negative cooperativity has been convincingly disproved by Szabo et al. using a chimera enzyme^16^. Furthermore, as will be discussed below, the heterogeneity would correspond to populations in roughly 1:5 ratio between free: bound state if it had been attributed to binding, which would indicate an extremely weak disassociation constant, which would be highly sensitive to the ligand concentration. The observed heterogeneity was thus attributed to distinct conformations in slow exchange on the NMR time scale. The assignments of the *apo* form could be successfully transferred to the dUpNHpp *holo* form, and when doing so the assignment was annotated to the major resonance for the heterogenic peak.

**Fig. 7:**
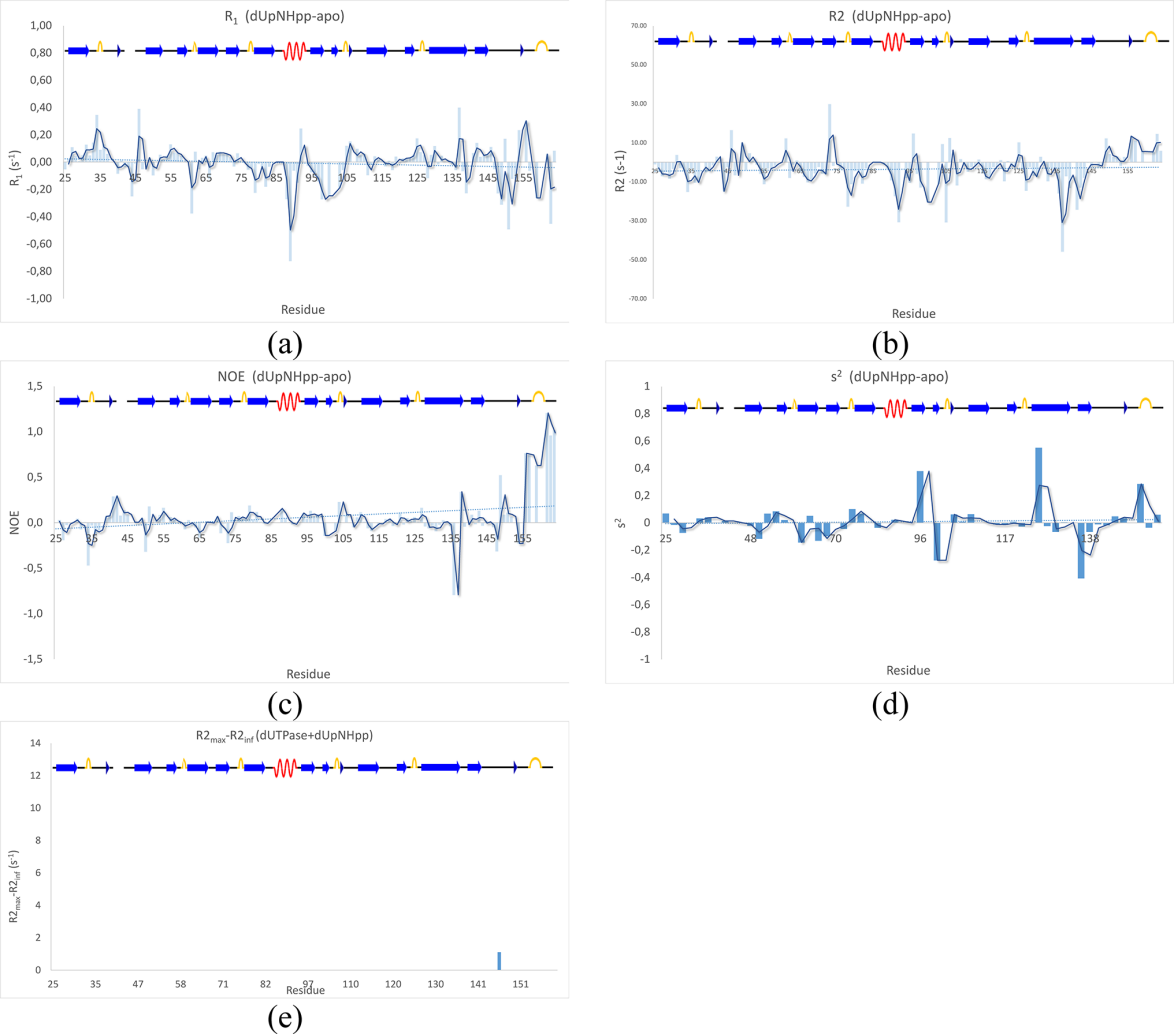
Difference plots of the relaxation parameters of dUpNHpp-Apo. (**a**) R_1_, (**b**) R_2_, (**c**) ^15^N-NOE, (**d**) S^2^ and (**e**) relaxation dispersion for the dUTPase + dUpNHpp sample (compare to Fig. 4f).

The substrate binding caused a slowing down of the millisecond to microsecond time scale dynamics observed for the *apo* form, into the milliseconds to seconds regime where multiple sub-conformations became individually observable. This was reflected by the R_ex_ contributions to the relaxation dispersions observed for the *apo* form were abolished upon substrate binding (Fig. 7e), except for the single residue, Q146.

The binding of the modified substrate had substantial effects on the relaxation parameters, as shown in the difference plots in Fig. 7. The clearest change is a stabilization of the C-terminal arm, reducing the degree of negative NOE for residues G156-N164. These residues interact with the triphosphate of the substrate, partially stabilizing the closed conformation of the C-terminal arm. There was also a systematic reduction of R_2_ upon substrate binding, likely attributed to the reduction of R_ex_ contributions from intermediate conformational exchange, as the enzyme backbone is stabilized. The differences in the order parameters (Fig. 7A-D) had a few hotspots along the sequences that the generated difference plots didn’t necessarily captures because of some residues being undetectable after substrate binding, thereby giving a zero contribution to the plot. For example, the R_1_ of the C-terminal arm is substantially changed for residues L148, D149, T151, G154, G156, G159 and K163, but in addition to that, residues S155, F158, S160 and T161 become undetectable, and does therefore not contribute to the difference plot. Two residues, G154 and G156, go against the trend and are slower for the *apo* form, but these resonances are also of very poor quality in the substrate bound form with big uncertainties in the integration. For example, the R_1_ of G154 is 1.22 ± 0.06 for *apo* and 1.45 ± 0.23 for *holo*. The R_2_ rates follows the same trend for the C-terminal, and together with the NOEs consistently shows that the C-terminal arm behaves more like the well-folded core of the protein in the substrate bound form than in the *apo* form, reflecting that the closed form of the C-terminal arm is stabilized by substrate binding.

One more patch shows changes in both R_1_ and R_2_, comprising residues A98, V100, E103, with G99, I101 and D102 becoming undetectable upon binding. In this case however, both the R_1_ and R_2_ rates become slower upon substrate binding, suggesting multiple time scales being involved. The R_2_ heatmap largely corresponds to the relaxation dispersion heatmap, in that the R_ex_ contributions from the slow ms-µs motions are lost when the substrate stabilize the enzyme. This patch is directly involved in the substrate-protein interactions and is also part of conserved motif 3.

The raw relaxation parameters are supplied in the Supporting information, Figure S2.

**Fig. 8:**
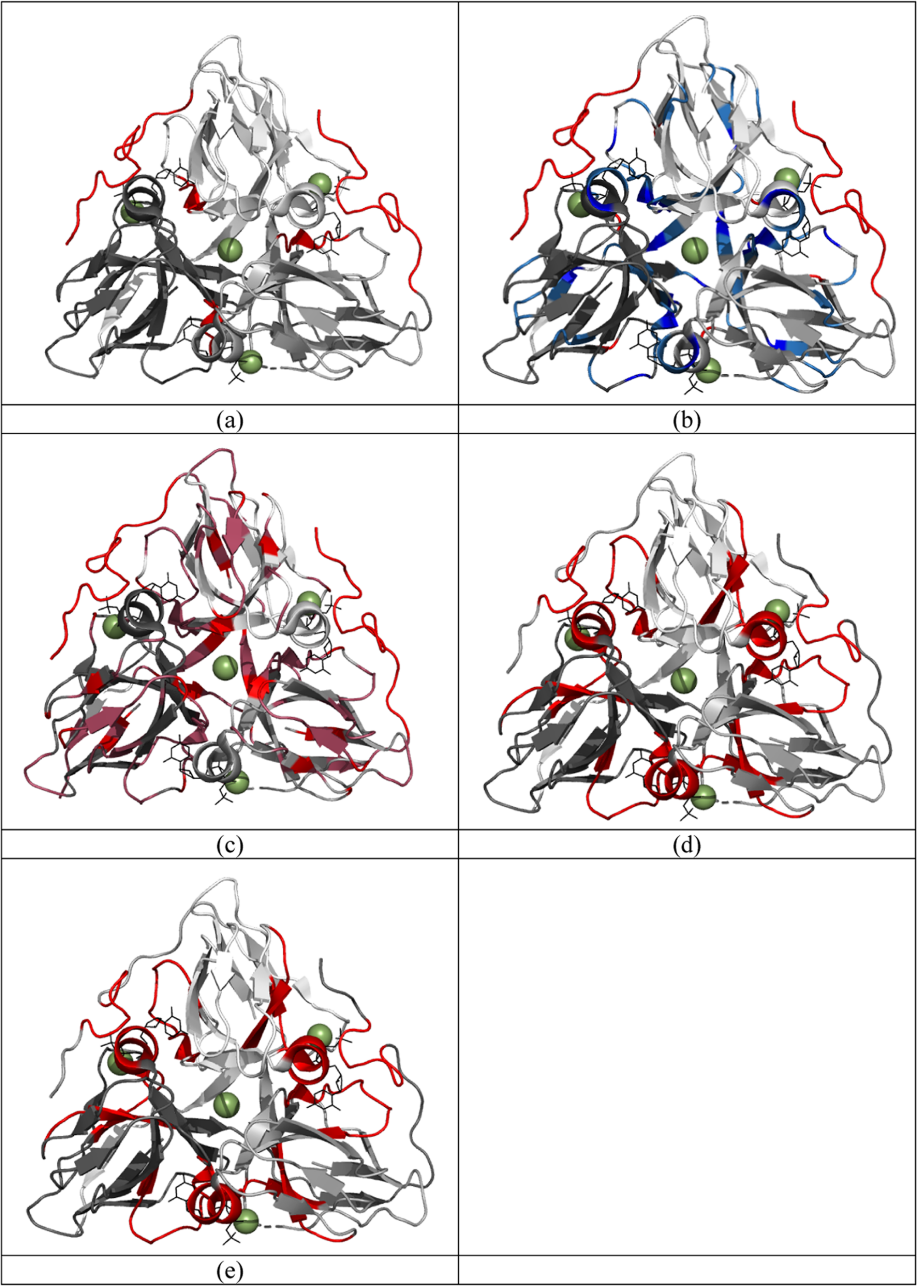
Heatmaps visualizing the hot spots for changes in (**a**) R_1_, (**b**) R_2_, (**c**) S^2^, (**d**) relaxation dispersion (cpmg) and (**e**) conserved motifs.

### Conserved motifs

It is well established that dynamics play a role in ligand recognition^40^. Generally, if certain types of dynamics are important for function, one would expect these to coincide with conserved parts within a protein family. One finds examples of such conservation in e.g. the NAGK protein family, where normal mode analysis indicates that slow movements of key residues are conserved^41^. Recent relaxation dispersion experiments have also successfully informed which residues are important for both function and dysfunction where dynamics are key^42,43^. One interesting observation is that the conserved Motifs 1–5 are all represented in the relaxation dispersion heatmap, reflecting that these conserved patches often display structural dynamics in the ms-µs timescale (Fig. 8). The secondary structure content of these motif includes mostly beta-strands (Motif 1 and 4), helical (Motif 2) and mostly loops (Motif 3 and 5). More generally, the unstructured C-terminal contains two conserved motifs, Motif 4 and Motif 5, the latter which comprise residues participating in binding interactions with the triphosphate of the substrate. Notably, there is a sequence segment, VVKTDI in the human sequence that resides between Motif 1 and Motif 2 which displays the characteristic ms-µs dynamics, and also has a quite high conservation. Conversely, many structured parts of the sequence are not conserved, and does not take part in slow dynamics. Taken together, this strongly suggests that essential features of dUTPase is correlated to its structural dynamics, both in the binding and release of substrate, and in the structural rearrangements involved in catalysis.

### Asymmetry between subunits and homotropic allosterism

It has previously been suggested that dUTPase displaying non-linear spectral response to ligand titration is the result of homotropic allosterism^10^, where a binding event in one subunit cause a change in ligand affinity in another subunit of the homotrimer. This has later been convincingly refuted by the construction of chimera trimers^16^. A possible explanation for the observed unexpectedly efficient quenching of the NMR signal intensity upon ligand binding is that intermediate exchange between bound and free ligands, and/or slow exchange between conformations in the bound state before the binding sites are saturated, contributes to the relaxation by a *R*_*ex*_ term, making the system deviate from a 2-state model and thereby giving the impression of allosteric effects even in its absence. The observed heterogeneity upon dUpNHpp binding displayed several species in complex ratios (Fig. 6a-c) but this does not imply allosteric effects. The observed heterogeneity is not concentration dependent, which means that the observed heterogeneity represents binding saturation at equilibrium. The most plausible explanation for this observation is that the internal dynamics of the protein gets slowed down while also becoming trapped by the uncleavable substrate in the conformational isomerization step, previously suggested in the kinetic model by Toth et al.^12^. This would make multiple species individually observable on the NMR time scale, in populations that results from the k_1_ and k_-1_ of the conformational equilibrium prior to the catalytic step, together with the possible combinations of states in the three subunits. The kinetic model suggests 21.2 Hz for the forward process and 3.7 Hz for the backward process. If we approximate that to a 5:1 ratio for simplicity and combine it with the possible eight states of the three subunits, that reduces to 1:3:3:1, we get a combination of 1:3:3:1 with 5:1, which would translate to 1:12:48:64 ratio of the populations. In other words, we would expect to find two major peaks, one slightly more intense than the other, and one, possibly two, minor peaks (64:125 and 48:125 of the total residue signal respectively), one being very weak, per residue if the spectra corresponded to the expected populations based on the kinetic model where the chemical step has been blocked. Several residues in Fig. 6a-c indeed fits this pattern, for example residues G80, A83, V109 and G126. This lends a different kind of support to the suggested kinetic model, while also providing a plausible explanation for the observed heterogeneity upon dUpNHpp binding.

### Hydration

**Fig. 9:**
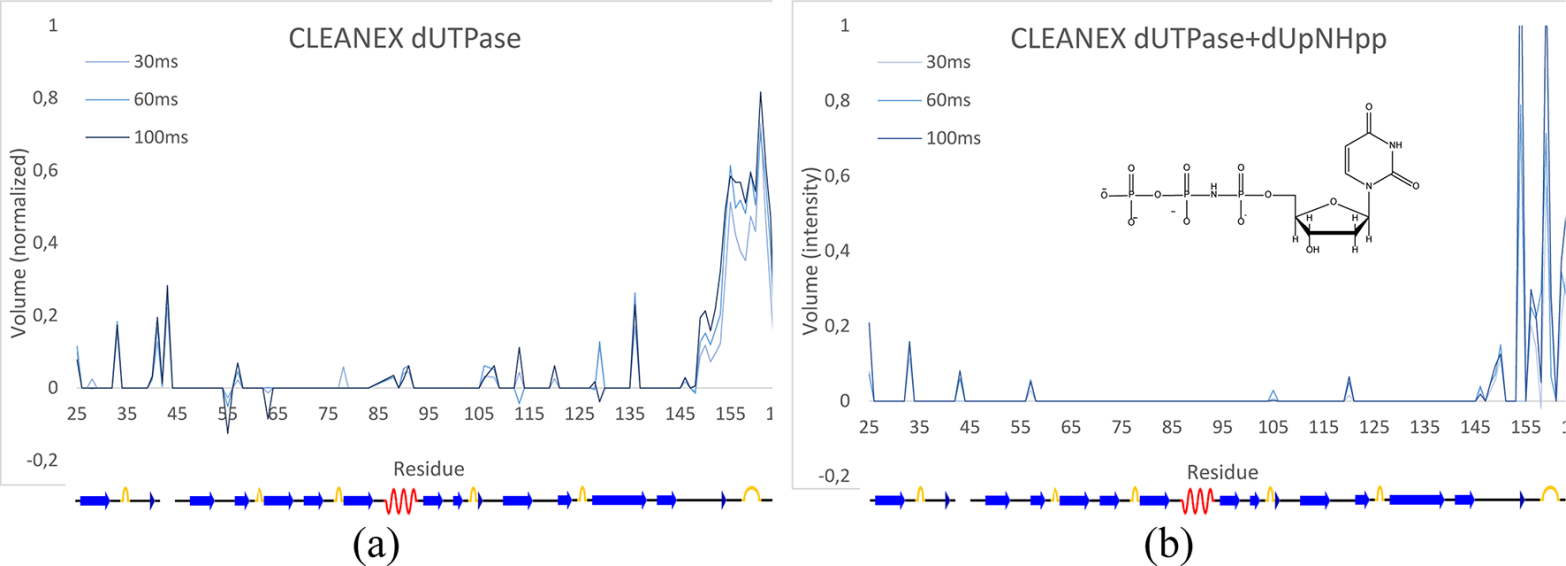
Water exchange between the backbone amide protons and the bulk water (**a**) without- and (**b**) with the bound substrate analogue, dUpNHpp.

The stabilization of the C-terminal arm as well as several core residues can also be observed as a reduction in amide proton exchange with the bulk water, detected by the CLEANEX-PM experiment (Fig. 9). This is the result of these residues being less exposed to the bulk solvent, again reflecting a stabilization of the closed form by the substrate binding. The same observation can be made for residues A83, A89, A90, Y105, R106 and V112, in the active site, belonging to the conserved motifs 2 and 3, as well as D127, R128 and E135 belonging to the conserved motif 4.

**Fig. 10:**
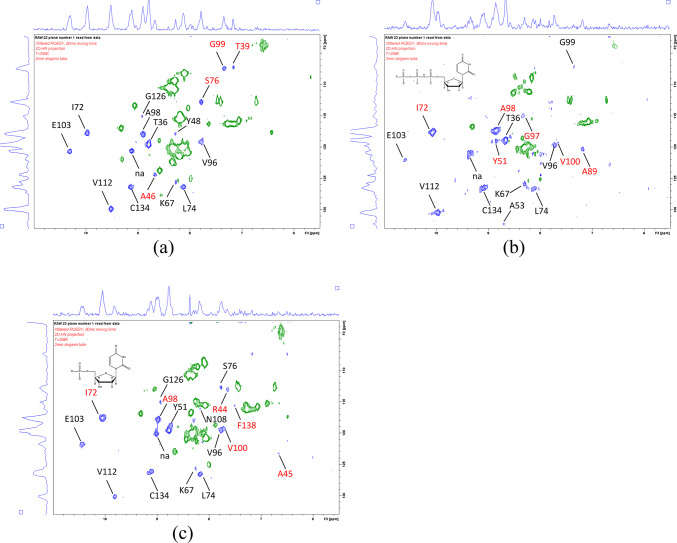
Water molecules with a residence time longer than the ns scale can transfer signal to amide proton on the backbone via cross-relaxation, here detected on the water plane of the NH projection of an x-filtered 3D ROESY-HSQC (blue) (60 ms spinlock) for (**a**) apo enzyme, (**b**) dUTPase + dUpNHpp and (**d**) dUTPase + dUMP. The exchange peaks between bulk water and the amides have opposite sign (green).

Changes in the dipole-dipole ROESY peaks to the bulk water, with opposite sign to the bulk water exchange peaks, were also observed (Fig. 10). In the presence of the modified substrate, dipole-dipole ROEs could be detected for residues G97, A98 and V100, which were either absent or weaker for the *apo* form. These residues’ amide protons are close in space to the catalytic water that is coordinated by V100 and the sidechain of the strictly conserved D102. This suggests that not only the enzyme backbone dynamics is stabilized by substrate binding, but also some of the bound water molecules increase their residence time.

## Conclusions

The dynamics study of human dUTPase is the first complete characterization of its structural dynamics. The results painted a comprehensive picture of the complex dynamics of the apo form, allowing it to bind the substrate before the active site closes by placing the c-terminal arm over the active site, thereby reducing the conformational freedom of the otherwise flexible c-terminus. A corresponding reduction in slow conformational exchange rates was observed for the entire enzyme upon substrate binding, where the “uncleavable” substrate, dUpNHpp, trapped the enzyme in the state before the chemical reaction. A structural isomerization step has previously been suggested in the literature, prior to catalysis, which is also what we believe that we observe in the holo form. The slowed down conformational exchange leads to the observation of several subspecies, owing to a slow equilibrium between two major conformations before catalysis at different populations, combined with 3 independent subunits, resulting in a complex pattern of resonances for the holo form. These results are in excellent agreement with previously published kinetic models of human dUTPase catalysis, and offers the dynamics resolution of individual residues for a comprehensive description of enzyme behavior in the presence and absence of substrate and product.

## Electronic supplementary material

Below is the link to the electronic supplementary material.

Supplementary Material 1

## Data Availability

The structural dynamics integration data is available at the Zenodo depository: https://zenodo.org/doi/10.5281/zenodo.13235592.

## Abbreviations

dUTP: Deoxyuridine 5’-triphosphate
dUTPase: Deoxyuridine 5’-triphosphate nucleotidohydrolase
NMR: Nuclear magnetic resonance
dUPNPP: 2’-Deoxyuridine 5’-α,β-imido-triphosphate
