# Supplementary material for: Structural dynamics of human deoxyuridine 5’-triphosphate nucleotidohydrolase (dUTPase)
Source: Sci Rep. 2024 Oct 30;14:26081. doi: 10.1038/s41598-024-76548-x (PMC11525568; doi:10.1038/s41598-024-76548-x)
Supplement: Supplementary file 1 — Supplementary Material 1 [file 41598_2024_76548_MOESM1_ESM.docx]

Structrual Dynamics of Human deoxyuridine 5'-triphosphate nucleotidohydrolase (dUTPase)

Ravdna Sarre^a^, Olena Dobrovolska^b^, Patrik Lundström^c^, Diana Turcu^b^, Tatiana Agback^d^, Øyvind Halskau^b^, Johan Isaksson^a,e*^

^a^Department of Chemistry, UiT the Arctic University of Norway, Box 6050 Langnes, 9037 Tromsø, Norway

^b^Department of Molecular Biology, University of Bergen, Box 7800, 5020 Bergen, Norway

^c^Department of Physics, Chemistry and Biology, Linköping University, 581 83 Linköping, Sweden

^d^Department of Molecular Sciences, Box 7015, SE-750 07 Uppsala, Sweden

^e^Department of Pharmacy, UiT the Arctic University of Norway, Box 6050 Langnes, 9037 Tromsø, Norway

^*^To whom correspondence should be addressed. Email, [johan.isaksson@uit.no](mailto:johan.isaksson@uit.no)

**
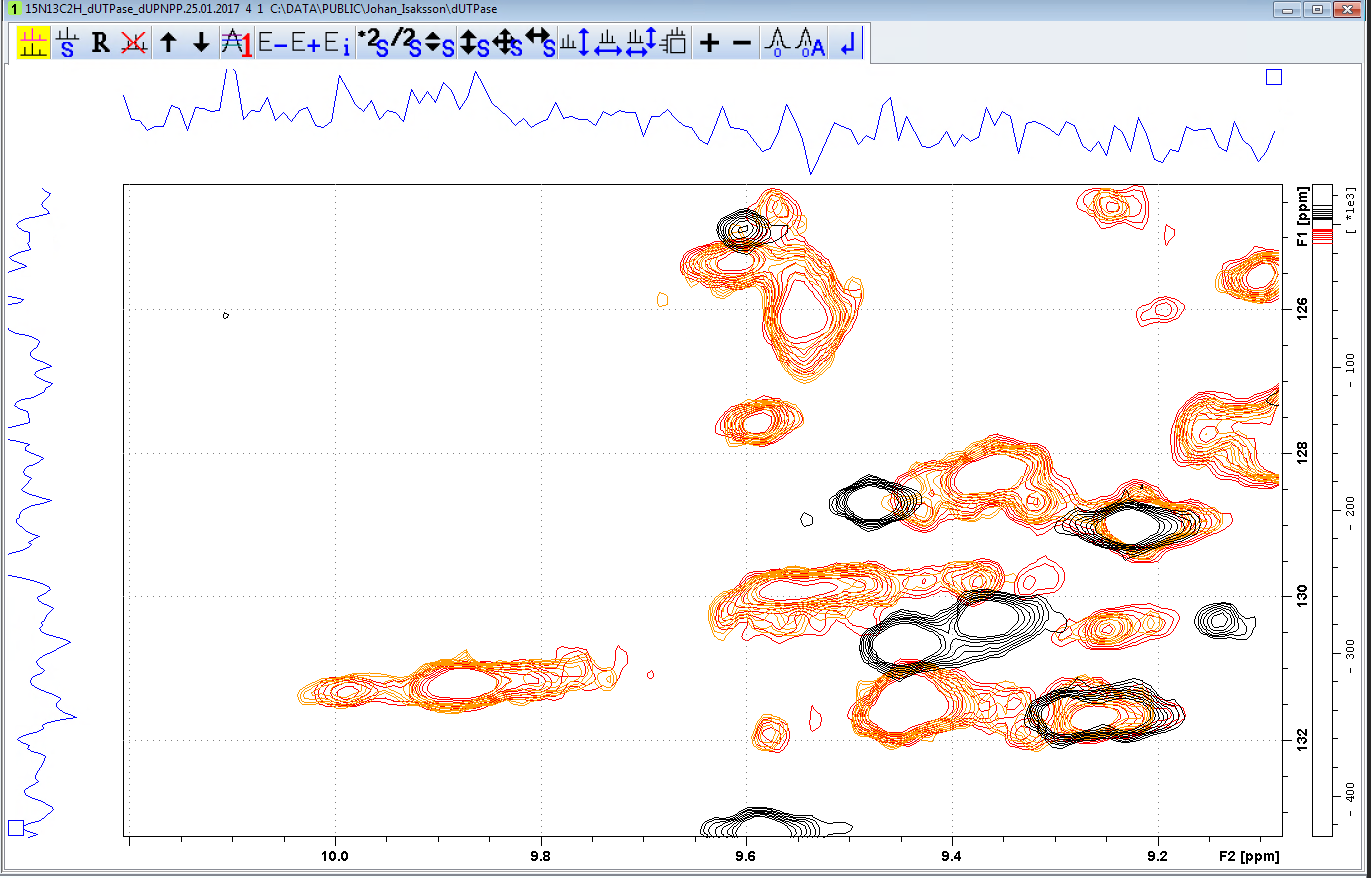
**

**Figure S1.** Superimposed NHSQC spectra of dUTPase (black) (apo), dUTPase+dUpNHpp (red) (20ul ligand stock) and dUTPase+dUpNHpp (orange) (40 ul ligand stock), showing that the heterogeneity of the holo form is not dependent on the ligand concentration.

|  |  |
| --- | --- |
| (a) | (b) |
|  |  |
| (c) | (d) |

**Figure S2**. Relaxation parameters, (a) R_1_, (b) R_2_, (c) ^15^N-NOE, (d) s^2^ calculated with Modelfree at 600 MHz fitting s^2^ and t_e_, (model 2 in the Modelfree documentation). All experiments were acquired at 37 ºC.

| 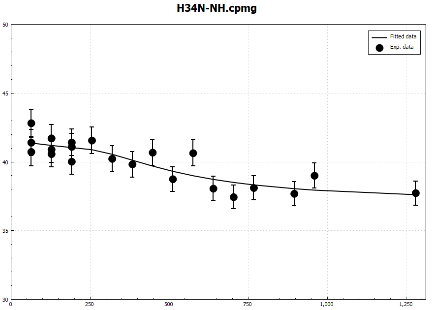 | 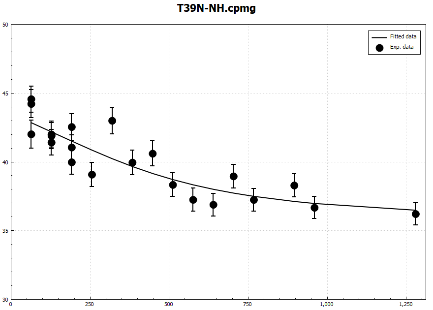 | 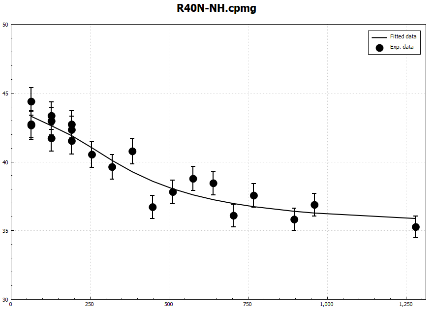 |
| --- | --- | --- |
| 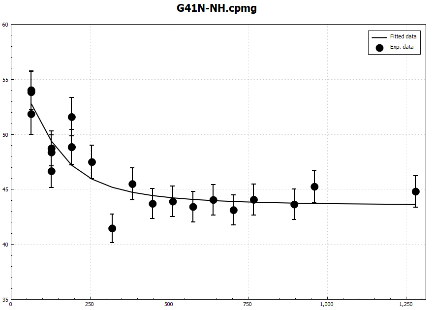 | 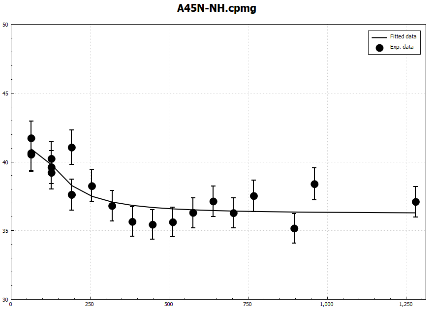 | 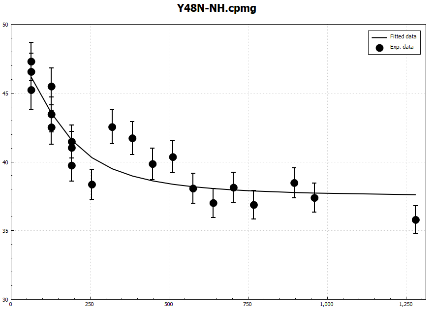 |
| 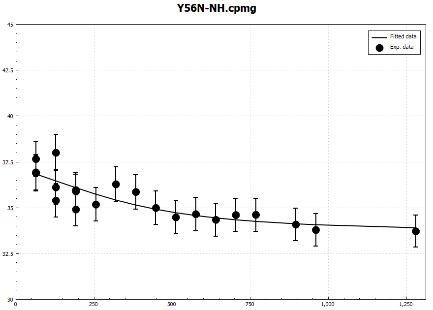 | 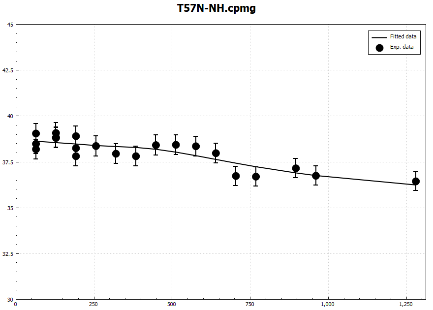 | 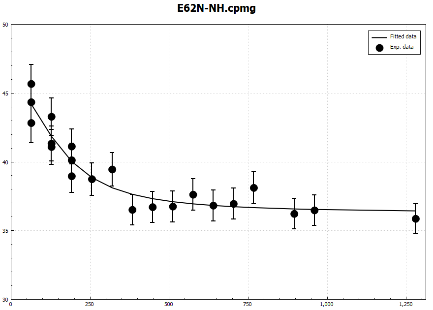 |
| 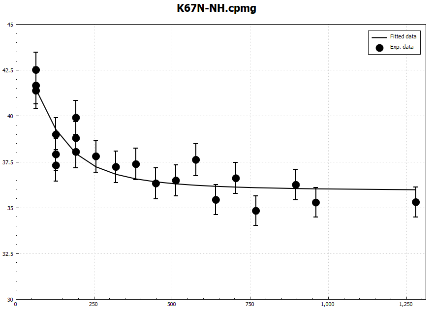 | 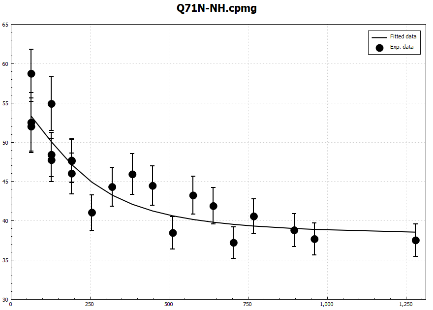 | 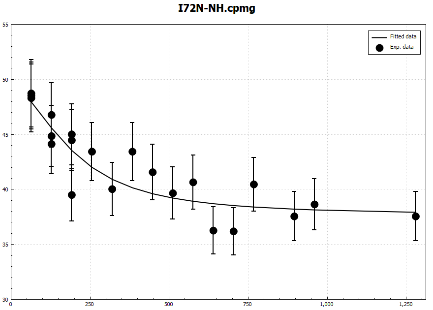 |
| 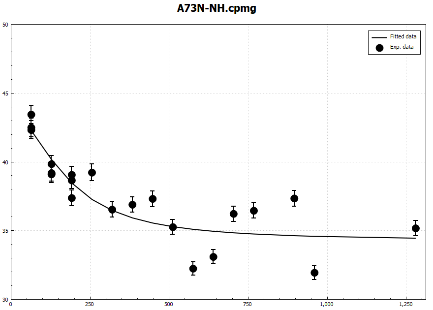 | 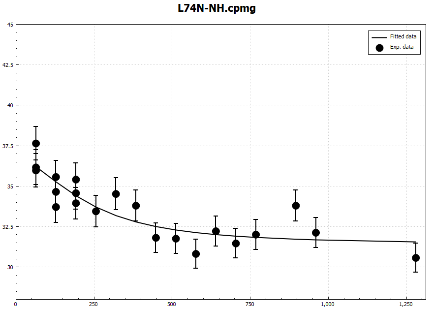 | 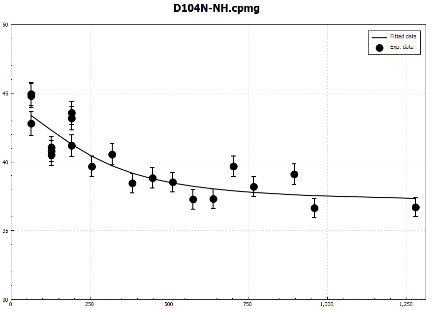 |
| 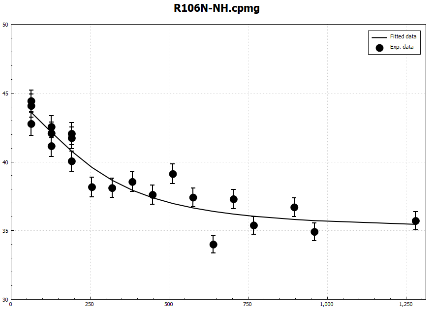 | 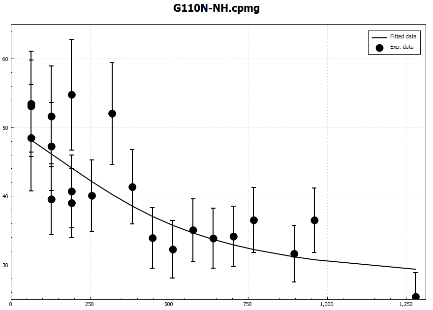 | 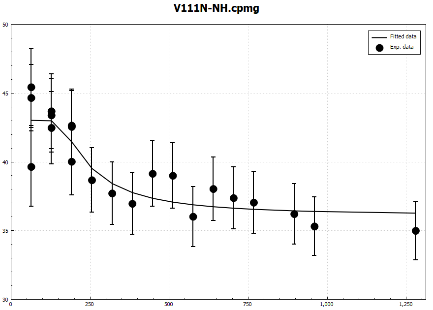 |
| 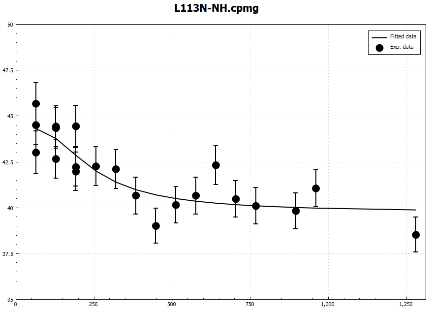 | 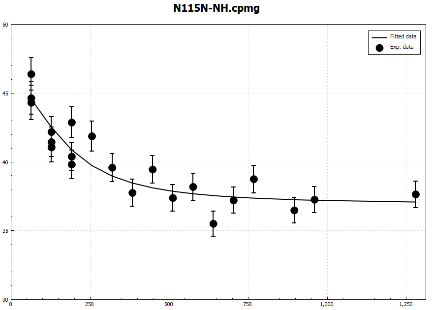 | 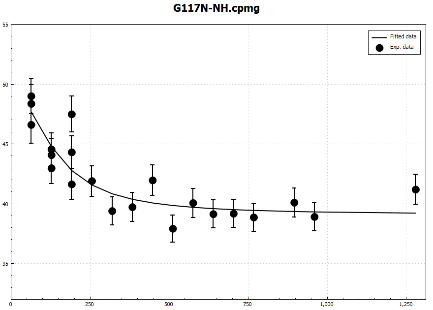 |
| 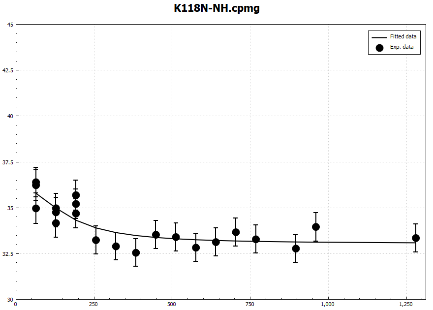 | 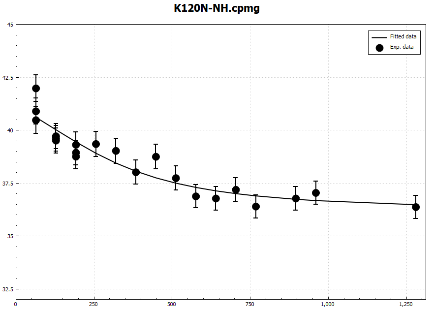 | 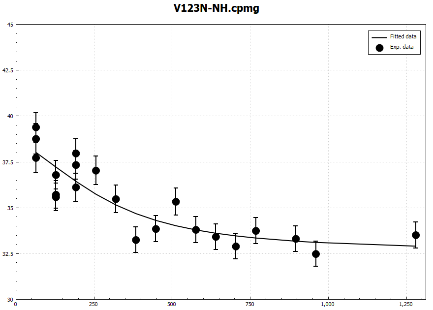 |
| 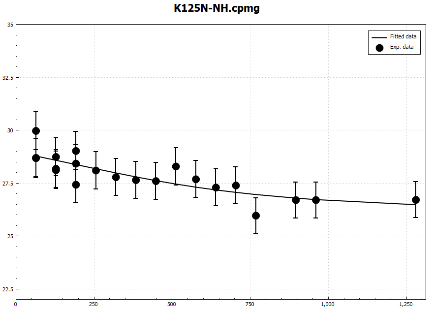 | 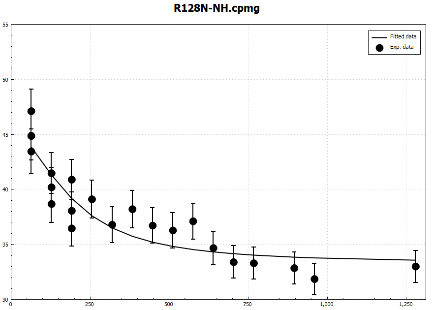 | 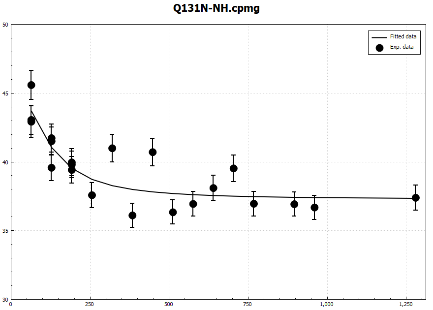 |
| 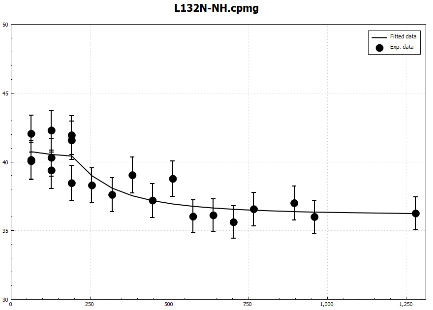 | 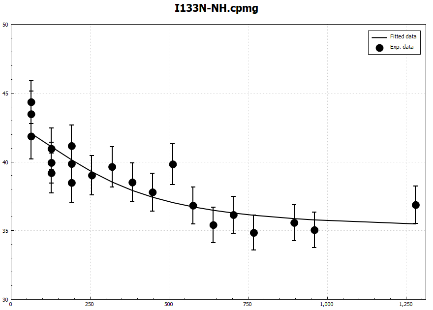 | 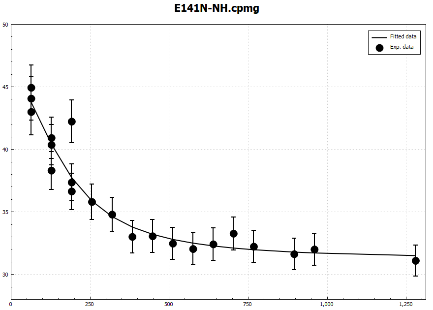 |
| 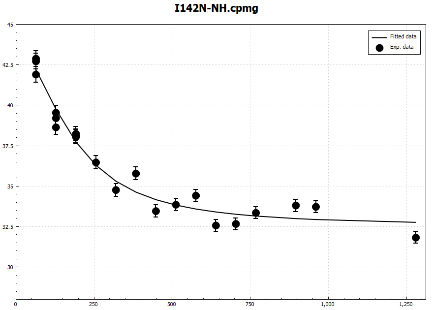 | 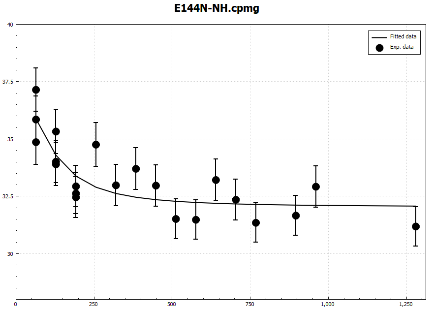 | 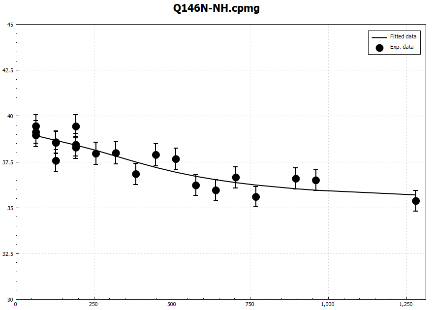 |
| 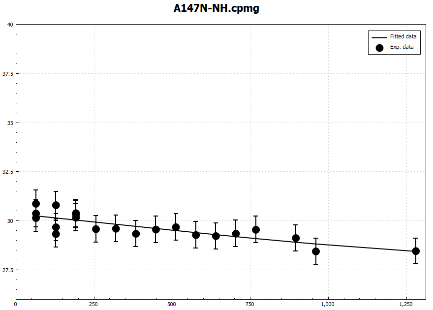 |  |  |

**Figure S3**. Relaxation dispersion profiles of the residues with a p-value cut-off of 0.1 for the dUTPase apo form acquired on a 600 MHz Bruker Avance III HD spectrometer at 298 K, integrated and plotted in PINT.

**Figure S4**. Correlation between the fitted chemical shift difference between the resting state and the “excited” state of the apo form with the chemical shift difference between the apo and holo forms.
